# Delayed Bleeding of Coronary Artery after Thoracoscopic Intradiaphragmatic Bronchogenic Cyst Resection

**DOI:** 10.3779/j.issn.1009-3419.2018.08.12

**Published:** 2018-08-20

**Authors:** Yuanda CHENG, Yang GAO, Abdillah N. JUMA, Chunfang ZHANG

**Affiliations:** 1 Department of Thoracic Surgery, Xiangya Hospital, Central South University, Changsha, China; 2 Department of Surgery, Kilosa Clinical Offficer Training College, Morogoro, Tanzania

**Keywords:** Bleeding, Coronary artery, Intradiaphragmatic bronchogenic cyst, Troca, VATS

## Abstract

Bronchogenic cyst occurring in the diaphragm is rare and thoracoscopic cyst resection is mainly effective treatment. The coronary artery bleeding after video-assisted thoracoscopic surgery (VATS) has never been described; here we report a case of left coronary artery injury after thoracoscopic itradiaphragmatic bronchogenic cyst resection, which may be caused by metallic troca or chest tube.

## Introduction

Bronchogenic cysts are congenital abnormalities, which are often located in the posterior mediastinum and rarely occur within the diaphragm. Up to June 2015, there were 20 cases of intradiaphragmatic bronchogenic cysts reported in the English literature^[1]^. It may present without any specific symptoms. Intradiaphragmatic bronchogenic cysts are easily mistaken to be abdominal or thoracic masses before surgery. Active bleeding after thoracic surgery is a common complication, but coronary artery bleeding after video-assisted thoracoscopic surgery (VATS) is extremely rare. We present a case of delayed coronary artery bleeding after thoracoscopic intradiaphragmatic bronchogenic cyst resection, which may be caused by metallic troca in VATS or chest tube after surgery.

## Case report

A 51-year old man presented with one-month history of chest pain. There was no history of any surgery or trauma. Family history was unremarkable. Thoracic computed tomographic (CT) scan revealed an ovoid soft tissue mass in the left posterior costophrenic angle, measuring about 4.3 cm×2.6 cm×5.8 cm (Fig 1A). The mass was resected under VATS, intraoperatively, it was found that the mass was closely located to the diaphragm; initially it was diagnosed as neurogenic tumor of the mediastinum but the final pathology was bronchogenic cyst (Fig 1B). The operative course was uneventful and a chest tube was inserted through the camera port at the 7^th^ intercostals space midaxillary line. Nothing special was noted on the first day after surgery, however, on the second day the patient with two episode of hypotension which was treated by fluid expansion since there was no evidence of active bleeding from the chest tube. Suddenly, patient presented with hemorrhagic shock and cardiac arrest as evident of gush of blood from the chest tube, resuscitation and stabilization was done and the patient underwent emergent thoracotomy.

**1:**
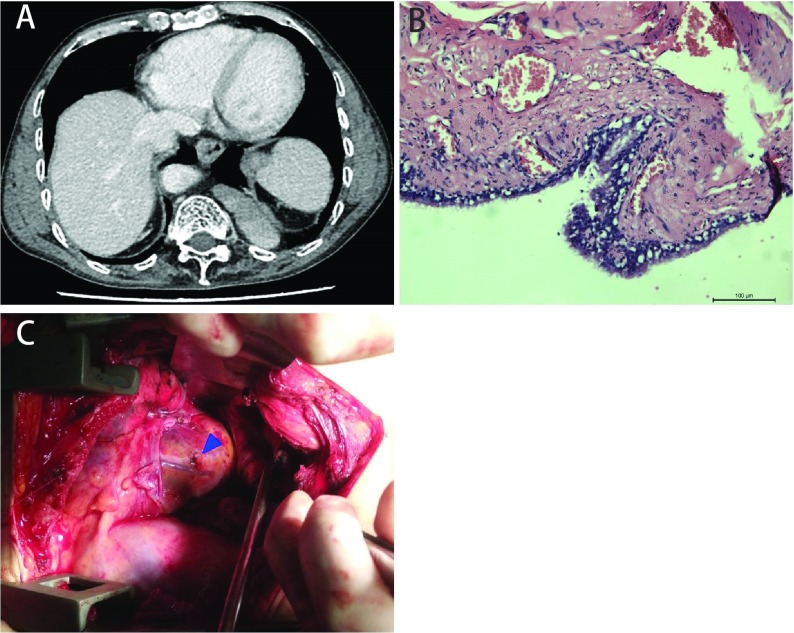
Thoracic CT, pathology, surgical image and simulated diagram of the patient. A: Thoracic CT scan revealed an ovoid soft tissue structure in the left posterior costophrenic angle. B: Bronchogenic cyst is diagnosed pathologically (HE staining, ×400); C: Intraoperative photograph showed the obtuse marginal artery from left coronary artery was damaged and bleeding, which was directly closed with 4-0 prolone.

After clearing the blood clots inside the chest, pericardium was distended by accumulated blood and further inspection revealed active bleeding coming from 3 mm hole on the pericardium. The pericardium was opened to relieved cardiac tamponade. The bleeding was found originating from injured obtuse marginal artery of left coronary artery. Because the injury was at the distal end of the obtuse marginal artery, it was directly closed with 4-0 prolene (Fig 1C). The patient successfully weaned from ventilator 2^nd^ postoperative day, and finally discharged from the hospital.

## Discussion

For the present case, what were the reasons of coronary artery bleeding and why it was not noted in the first day after surgery? Unfortunately, the operation video wasn't saved, so it is difficult to determine how the injury occurred during the operation.

Spontaneous coronary artery rupture (SCAR) with symptoms of sudden onset of chest pain and hemodynamic collapse has been previously reported^[2]^. SCAR is very rare and its etiology often includes atherosclerotic disease, aneurysm or dissection, trauma, and localized infection^[3-6]^. However, the medical history and the hole in the pericardium in the present case seem not to support the above possibilities. It is clear that coronary artery was not bleeding during the operation, but it may be injured without being noticed. Nevertheless, there is a great possibility of coronary artery injury during the surgery.

Owing to the barrel chest deformity as reviled by the thoracic CT, the camera port placement was very close to the pericardium which may in turn injured coronary artery. From the Fig 2A, the metallic sharp-edged troca can damaged the pericardium and coronary artery completely, when was inserted it into the thoracic cavity from the camera port. Another considerable cause of the coronary artery injury was the chest tube, the tip of which may be placed next to heart (Fig 2B). With the beating of the heart, the tip of chest tube repeatedly rubbed and stimulated the heart. When the position of the chest tube changes, the symptoms change as well, so two events of unexplained hypotension and left chest pain also supported this speculation. After coronary artery rupture, cardiac tamponade occurred and then cardiac arrest.

**2:**
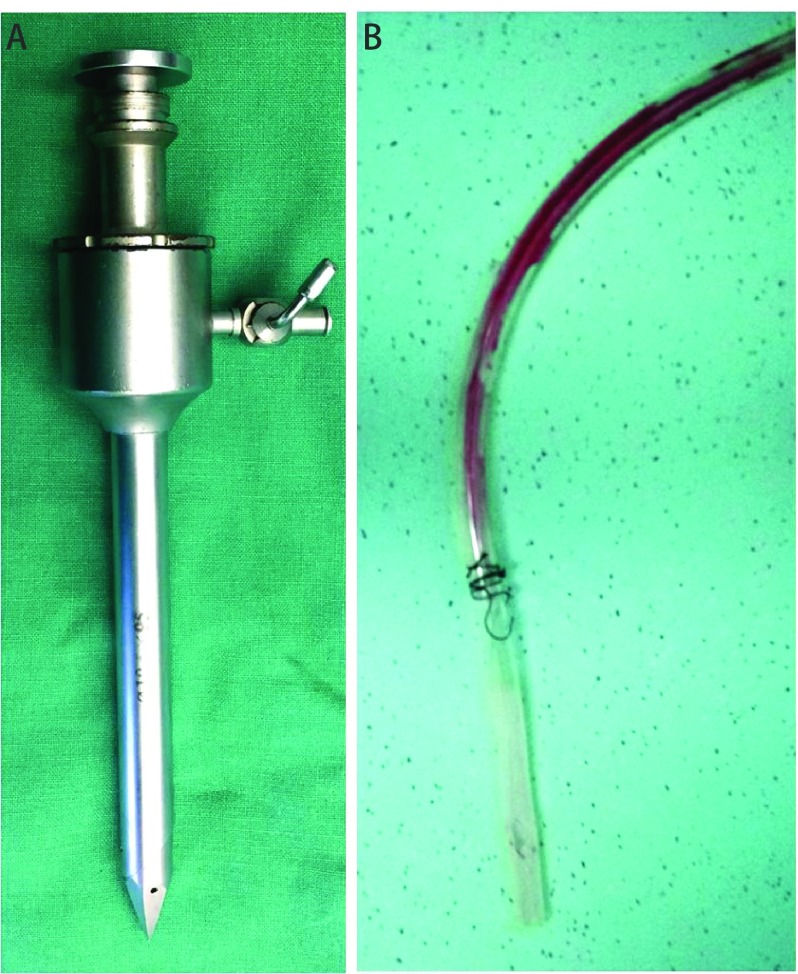
The metallic sharp-edged troca and chest tube

To prevent this complication, it is important to note that: Firstly, carefully thoracic examination including pericardium after VATS should not be neglected. Secondly, the use of any sharp-edged troca is should be discouraged in VATS procedures. Thirdly, in deformed chest the ports placement and incisions should be based on the thoracic CT scan. Lastly, the thoracic drainage tube should be noninvasive and placed in proper depth and position.

## Disclosures

The authors have no conflict of interests to declare.
